# Sensing Technology for Fish Freshness and Safety: A Review

**DOI:** 10.3390/s21041373

**Published:** 2021-02-16

**Authors:** Leonardo Franceschelli, Annachiara Berardinelli, Sihem Dabbou, Luigi Ragni, Marco Tartagni

**Affiliations:** 1 Department of Electrical, Electronic and Information Engineering, Guglielmo Marconi-University of Bologna, Via Dell’Università, 50, 47521 Cesena, Italy; marco.tartagni@unibo.it; 2 Department of Industrial Engineering, University of Trento, Via Sommarive, 9, Povo, 38123 Trento, Italy; annachiara.berardinelli@unitn.it; 3 Centre Agriculture Food Environment, University of Trento, Via E. Mach, 1, S. Michele All’Adige, 38010 Trento, Italy; sihem.dabbou@unitn.it; 4 Department of Agricultural and Food Sciences, Alma Mater Studiorum, University of Bologna, Piazza Goidanich 60, 47521 Cesena, Italy; luigi.ragni@unibo.it; 5 Interdepartmental Center for Industrial Agri-Food Research, University of Bologna, Via Q. Bucci 336, 47521 Cesena, Italy

**Keywords:** sensors, fish freshness, fish spoilage

## Abstract

Standard analytical methods for fish freshness assessment are based on the measurement of chemical and physical attributes related to fish appearance, color, meat elasticity or texture, odor, and taste. These methods have plenty of disadvantages, such as being destructive, expensive, and time consuming. All these techniques require highly skilled operators. In the last decade, rapid advances in the development of novel techniques for evaluating food quality attributes have led to the development of non-invasive and non-destructive instrumental techniques, such as biosensors, e-sensors, and spectroscopic methods. The available scientific reports demonstrate that all these new techniques provide a great deal of information with only one test, making them suitable for on-line and/or at-line process control. Moreover, these techniques often require little or no sample preparation and allow sample destruction to be avoided.

## 1. Introduction

Consumer acceptance and food safety are key concerns for wholesalers and retailers of fresh fish and seafood products [1]. Fish and fish products are appreciated worldwide and have a very important role in a balanced human nutrition, providing several different nutrients and health benefits (protein, omega-3 fatty acids, vitamins). However, fresh fish and fish products are highly perishable products and are subject to a fast development of undesirable odors and flavors, and to a rapid decay process. This is due to their biological composition and several physical and chemical characteristics: low content of connective tissue, presence of autolytic enzymes, neutral pH, and high-water activity [2]. The time occurring between production and spoilage, caused by biochemical reactions and microbiological activity, defines the shelf life of fresh fish [3]. Spoilage of fish is a process of deterioration in the quality of fish that changes its appearance, odor, smell, taste, and meat texture. After a short initial spoilage stage, the increase of pH and nitrogen substances causes a multiplication of microorganisms, influencing the color of the eyeballs, body surface, abdomen, and muscle tone [4].

Reliable methods and techniques are required to evaluate the fish quality and spoilage [5]. Sensory attributes are the principal parameters of fish quality, and are related to fish appearance, color, meat elasticity or texture, odor, and taste. Based on changes in these variables, a series of sensory attributes like the brightness of appearance, meat color, gill color, meat elasticity, and smell could be used to classify freshness [6]. The most common sensory evaluation technique is the quality index method (QIM), based on the assessment of sensory quality parameters regarding mainly the skin, eyes, gills, and abdomen of raw fishes. This method, developed for each fish species, assesses parameters that significantly change during the shelf life and it is extensively used to estimate fish quality, although the support of other methods is required [7].

Quantitative chemical and physical techniques are correlated with sensory parameters to evaluate fish freshness and quality attributes. Chemical attributes are related to biochemical changes that occur in a fish after the harvest. Some of the most used parameters are specific spoilage organism (SSO) growth, lipid oxidation, peroxide value (PV), and the K value, based on adenosine triphosphate (ATP) degradation [8]. Physical properties like texture, electrical, and optical parameters could be used to assess the fish freshness [9,10,11]. Chemical and physical attributes correlate well with sensory attributes but can be affected by several factors, such as species, age, proximate composition, fishing area, season, and animal nutritional status [12,13].

The cited chemical and physical quantitative techniques have plenty of disadvantages, as they are destructive, expensive, time consuming, and require highly skilled operators. Moreover, they cannot be applied on-field or in on-line detection. Additionally, the fact that they are very time-consuming is detrimental, because fishes must often be put in the market the same day they are killed, given the fast spoilage time. The more innovative sensing techniques could overcome these problems, being rapid, cost-effective, and, except for biosensors, not invasive, permitting on-line detection to be achieved [5].

**Figure 1:**
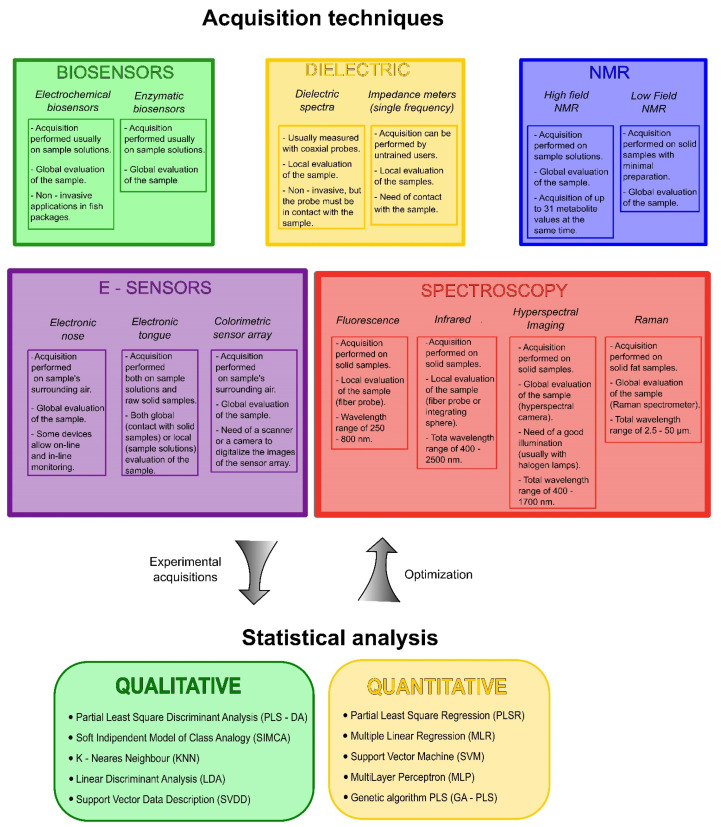
Summary scheme of the indirect acquisition techniques reported in the paper (on the left) and of the different statistical algorithms used for the creation of a calibration model (on the right).

The review presents and describes the state of the art and future perspectives of these indirect techniques. Particularly, the text covers: biosensor techniques, e-sensing techniques, methods based on the fish dielectric properties, nuclear magnetic resonance (NMR), and spectroscopic techniques, including fluorescence, infrared, Raman, and hyperspectral imaging. Moreover, the review also covers the statistical analyses utilized in most of the presented papers. These algorithms, mainly utilized for the electronic and spectroscopic techniques, have a great and increasing importance in the sensors field, because they allow experimental data to be processed to create qualitative and quantitative models, enabling a fast and non-invasive measurement process. Almost all these algorithms are based on principal component analysis (PCA): it changes the original data space (defined by the measured variables) to a new space defined by the directions that contain the majority of data variance, called principal components (PCs). This analysis greatly reduces the data dimensions (usually from hundreds or thousands of variables to 5–10 PCs) and allows a better visualization of clusters and trends in the data. Evolutions of PCA allow the creation of both classification and regression models, such as the soft independent model of class analogy (SIMCA) algorithm, which applies PCA to different classes of data and then predicts the class of new measurements [14]. Regarding regression tools, the most used technique in chemometrics is partial least square regression (PLSR), developed by Wold in the late 1960s. It finds directions in the space (called latent variables) that explain at the same time the variance of both the experimental data and measured variable of interest [15]. Moreover, in the last years, algorithms like artificial neural networks (ANNs) were applied to nearly every field of food science, thanks to their high computational power and a consequent ability to handle more complex tasks [16]. The reported techniques are summarized in Figure 1, as well as the main statistical analyses used in these studies. The image also shows some of the most important practical characteristics of every method.

## 2. Analytical and Chemical Biosensors

Biosensors are devices able to measure a chemical or a biological reaction and to convert the response into an electrical signal [17]. These devices are functionally integrated and provide, through a biological recognition element, a (bio)transducer that recognizes the presence of a specific analyte in the reaction [18]. The signal generated by the biotransducer (based on heath absorption or light absorbance or redox reactions, or changes in the mass of reactant or products, etc.) produces changes in the electrical or electronic output that are transformed into a measurable signal by the transducers. The detector, finally, amplifies and processes the transduced signal [19]. According to the transducers, these devices can be classified as electrochemical, optical, thermal, and piezoelectric biosensors [20].

Concerning fish applications, electrochemical biosensors have been investigated to assess the presence of some compounds derived from the deterioration process. Between these compounds, the main ones are purine based like xhantine (Xa), produced by the degradation of adenosin-triphosphate (ATP); uric acid (UA) and hypoxhantine (HxA), both products of the action of xanthine oxidase on Xa; and gas molecules like trimethylamine (TMA) and dimethylacetamide (DMA), created by the conversion of trimethylamine oxide (TMAO) in fresh meat by spoilage bacteria, which also decompose urea and amino acids into ammonia.

Research performed by Tang et al. [21] showed that a sensitive amperometric sensor for xanthine can be created by modifying a carbon paste electrode with an electrospinning technique, obtaining carbon nanofibers (CNFs). Amperometric biosensors are based on the measurement of the electronic current produced by oxidation or reduction, and the use of nanomaterial greatly enhances the transduction of the biochemical reaction [22,23]. The CNF shows high catalytic properties towards the oxidation of Xa. The system was tested with crucian carp samples and the main results evidenced a linear relation between the measured current and the xanthine concentration, with a correlation coefficient of 0.99 and a limit of detection (LOD) of 20 nM.

To obtain a non-invasive measure of the content of ammonia in fishes, Heising et al. [24] monitored the changes of the ammonium content in an aqueous phase positioned in the headspace of the fish package, thanks to an ammonium selective electrode that measures changes in the electrical potential of water. Results showed that the trend of NH4+ measured in the aqueous phase was comparable with the trend of total volatile basic nitrogen (TVB-N) measured in the fish body. Two years later, Heising et al. [25] also investigated the use of simple and cheap electrode sensors, monitoring changes in the pH and conductivity of the aqueous phase. These electrodes are less selective with respect to the ammonium ones, but they can measure a broad range of substances related to the fish spoilage process. Results evidenced that the pH sensor could not be considered a good predictor of the overall sample quality, because the changes in the signal are too temperature dependent, whereas the conductivity sensor presented a pattern correlated with the increasing volatile amines content at all temperatures. More recently, Chang et al. [26] developed an amine gas sensor able to detect volatile amine gases in about 60 s from raw fish flesh. The gas sensor is based on a porous metal electrode, put on top of an organic semiconductor diode with nanostructures that increase the surface to volume ratio and therefore increase sensitivity. The fish samples were put in a bag of air, which was consequently dried and pumped through the sensor chamber, measuring the relative electric changes of air due to the increasing presence of ammonia, TMA, and DMA. Mackerel samples were used to confirm that the system was able to detect the expected influences of storage time, storage temperature, and different fish parts. Tests on tilapia and beltfish samples, compared with a traditional analysis of TVB-N, showed linear relations, with regression coefficients of 0.93, 0.96, and 0.97, respectively.

A system based on differential pulse voltammetry (DPV) was proposed by Vishnu et al. [27] for the simultaneous detection of three purine bases: HxA, Xa, and UA. In this configuration, a series of regular pulses are superimposed on a potential linear sweep or stairsteps, measuring the consequent changes in the current. Under optimal conditions, the sensor showed a linear behavior in the range of 6–30 µM, 8–36 µM, and 3–21 µM, respectively, with a current sensitivity of 0.921 µA µM-1, 1.742 µA µM-1, and 0.499 µA µM-1. Five different fish species (barracuda, lady fish, mackerel, blue catfish, and channel catfish) were used to test the device, with calculation of the recovery value. Acquisitions were performed before and after the addition of a known amount of one of the three purines to test the ability of the system to correctly measure the variation. The recovery value was about 100% in most of the cases, with the difference from the real value being not higher than 5%. These differences were explained by the strong biological activity of some species, which can block the active sites of the sensor.

Starting from an electrode of copper phosphate, characterized by a rapid production time (within 20 min), Lee et al. [28] set up an electrochemical histamine sensor with an innovative single-step dipping and coating technique. The obtained device, with a nanostructure similar to flakes, was tested in a Na2HPO4 solution with pH 8.5, showing good selectivity, an LOD of 3.0 ppm, and a detection range of 5–500 ppm. From 5 to 100 ppm, a very good linearity was found, with a correlation coefficient r of 0.999. The device was tested on fresh and rotten fish samples and compared with the commercialized liquid chromatography-mass spectroscopy, obtaining a deviation less than 10% between the two measures of the histamine concentration. Another interesting work is represented by the research conducted by Li et al. [29]. The authors studied the effect of various amounts of triethylamine on copper nitrate—benzenetricarboxylic acid (Cu-BTC) frameworks on the simultaneous determination of Xa and HxA in fish samples. Results showed that triethylamine had significant effects on the morphology, active response area, and electron transfer ability of Cu-BTC frameworks, enhancing the device sensibility. Tests on fish samples showed a linear behavior between the concentration and the oxidation peak of the two substances, in the range of 0–8 µM for Xa and 0–10 µM for HxA.

A particular and well-diffused family of biosensors is the enzymatic biosensors, based on the measurement of the reaction between a substance of interest and an enzyme. In particular, amperometric enzymatic biosensors, which are composed of an enzyme as a biological substance and an electrode as a transducer, are one of the most popular biosensors. In general, an amperometric enzymatic biosensor is an analytical device that combines an electroactive substance of interest with an enzyme immobilized on the electrode. An electrical signal gives the information regarding the presence and concentration of the substance and the results are calculated by a standard curve [19]. For fish freshness evaluation, chemical metabolites are the main target, especially ones related to ATP decomposition. Xanthine oxidase (XOD), which is involved in the reaction chain of ATP disintegration, is the main recognized element [5]. Thandavan et al. [30] developed a biosensor based on nanoparticles of iron oxide, which was able to measure nanomolar concentrations of Xa, enabling early detection of fish spoilage. XOD was linked to the nanoparticles, causing the electroreduction of hydrogen peroxide. The detection limit was found to be 0.4 nM, as well as a linear increase of the amperometric response of the sensor in the concentration range of 0.4–2.4 nM. Narang et al. [31] set up a system based on XOD immobilized on a nanocomposite platform of titanium dioxide nanoparticles and multi-walled carbon nanotubes. The device was characterized by an LOD of 0.5 µM and a linear behavior of the peak current for a xanthine concentration range of 0.5–500 µM, with a response time of about 30 s. Labeo fish samples were used to test the device, and a linear correlation (r = 0.99) with the Xa level measured by an enzymic colorimetric method was reported. Another example of amperometric enzymatic biosensors’ Xa application is represented by the work conducted by Borisova et al. [32] on hake samples. The system was composed of a glassy carbon electrode and a nanostructure sensing interface made by nanoparticles of polydopamine and platinum, where the enzyme of XOD was immobilized. This novel biosensor proved to have good reproducibility and repeatability, with a detection range of 50 nM–12 µM and an LOD of 13 nM. Additionally, Apetrei et al. [22] worked on a similar sensor for histamine biosensing, where the enzyme diamine-oxidase was immobilized on a carbon screen-printed electrode, modified with graphene and platinum nanoparticles. A low LOD (25.4 nM) and a linear range of 0.1–300 µM were observed in addition to correlations with the more traditional enzyme-linked immunosorbent assay (ELISA) method. More recently, Pierini et al. [33] used an edge-plane electrode made of pyrolytic graphite to determine Hx, Xa, and UA in four different kinds of fishes, thanks to the anodic stripping voltametric technique. This method is based on a stripping step, where the analyte of interest is oxidized from the electrode, and the resulting current is measured. A detection range of 0.1–50 µM for Hx and Xa and 0.1–25 µM for UA was obtained, alongside an LOD of 0.08, 0.06, and 0.03 µM, respectively. Linear regression equations for the net peak current in comparison with the analyte concentration were calculated, obtaining R2 values of 0.978, 0.992, and 0.990.

In 2018, Torre et al. [34] researched a biosensor based on a carbon electrode, where the enzyme diamine oxidase was immobilized. This simple architecture allowed a low cost and a construction time of only 30 min, obtaining, at the same time, a portable sensor for analysis of small volumes (30–40 µL). The histamine was measured through its electrochemical oxidation, thanks to the use of a redox mediator. The system, tested with spiked tuna and mackerel samples, provided a low LOD (0.97 mgL^−1^) and a good prediction accuracy, as well as a recovery value of 96–97%. Additionally, the stability of the system was investigated. Acquisitions were performed on stable histamine solutions, over a total of 35 days, obtaining a final maintenance of the original signal of 87.7%. Finally, Yazdanparast et al. [35] developed a sensor, based on a glassy carbon electrode, that allows detection of Xa thanks to a nano-biocomposite made by a multi-walled carbon nanotube and poly(L-aspartic acid) film that immobilizes XOD on the electrode. The system was tested on salmon fish samples, showing a low LOD (3.5 × 10^−4^ μM) and a linear behavior in two different ranges: 0.001–0.004 µM (R2 = 0.997) and 0.005–50.0 µM (R2 = 0.995). A recovery test was performed on fishes stored for 0, 2, and 8 days, obtaining mean recovery values that were always higher than 95%.

Instead of carbon nanostructures, platinum nanoparticles were used by Chen et al. [36] to mimic the catalytic activity of peroxidase in a fluorescence sensor, designed for the detection of HxA content in aquatic products. This method was tested with fish, squid, and shrimp samples, showing a detection range of 8–2500 μM and an LOD of 2.88 μM, much lower than the threshold specified by the national standard. Moreover, it has facile preparation, low cost, and high reusability.

Table 1 summarizes the presented biosensor techniques, focusing on the type of application (measured substance, fish species, place of application) and on the system results (LOD, detection range).

## 3. Electronic Multi-Sensory Techniques

As described above, sensory evaluation techniques, like QIM, are the most used methods to classify fish according to freshness [4]. However, these techniques do not seem to keep up with the increasing request of food and its consequent need for rapid quality assessments. According to this assumption, techniques able to simulate the human sensorial perceptiveness, such as e-nose and e-tongue, colorimetric sensor array, and e-eye, have seen an increasing consideration both in scientific papers and industrial applications, as shown in Table 2 [37]. All these devices are based on the use of several arrays of sensors, and the obtained data are often processed with statistical analysis.

### 3.1. Electronic Nose and Tongue

Electronic nose is a technique aimed at mimicking the functions of the human nose, not in the transduction principles, based on receptors, but in the use of arrays of biochemical transducers, followed by data processing. Within the variety of e-noses designed and created in the last two decades, we can observe systems based on electrochemical gas, metal oxide, and conducting polymer sensors coupled with different feature extraction and data processing methods.

A first application of the technique is represented by the prototype “FishNose”, from Haugen et al. [38]. It consists in a solid-state-based gas-sensor array system, set up to monitor the industrial smoked salmon process, thanks to the monitoring of parameters like TVC and lactic acid bacteria (LAB). Correct classification rates ranging from 93% to 95% were evidenced for fresh and aged samples, respectively.

TVC was also measured by a sensor based on four integrated metal oxide micro-sensors, developed for sardine discrimination according to different freshness stages (fresh, medium, and outdated) by using fishes stored for 2, 3, and 4 days, respectively [39]. More recently, Semeano et al. [40] monitored the spoilage of tilapia thanks an optical electronic nose, made with a gas-sensitive hybrid gel material, obtaining changes in the optical signals consistent with the reproduction of microorganisms. An e-nose system named “Mastersense” was developed by Grassi et al. [41], operating with four MOS sensors. Two different classification methods, K-nearest neighbor (K-NN) and partial least squares–discriminant analysis (PLS-DA), were developed to classify the freshness of samples using e-nose information, correctly classifying fish (plaice and salmon) samples into three freshness classes defined by TVC (unspoiled, acceptable, spoiled). Even though some samples belonging to the intermediate freshness level were misclassified, none of the samples with a risky microbial concentration were classified as being acceptable for consumption, revealing that the model can be safely used for real applications. Total volatile basic nitrogen (TVB-N) and aerobic bacterial counts were instead measured by Tian et al. [42] to study the effect of different storage temperatures (5, 10, and 15 °C) and storage times (3, 4, and 5 days) in hairtail fish, using a system based on metal oxide sensors. The collected data were used as input for a PCA analysis, for the compensation of humidity and temperature effects and the creation of calibration models. R^2^ values from 0.91 to 0.97 were observed for the two parameters. In the work by Liu et al. [43], the use of a self-designed e-nose system, known as “UTS NOS.E”, was united with a powerful statistical tool, the hidden Markov model (HMM), to establish freshness evaluation models for salmon fillets. Three different uses of HMM were discussed, all obtaining a sensitivity and specificity higher than 95%.

Electronic tongue refers to a device made of sensors that, thanks to the transduction of a signal or a pattern of them, permits identification of tastes in (soluble) food, obtaining a rapid assessment of complex liquids, whereas the e-nose described above works with gaseous systems. In the literature, it is possible to find different electrochemical sensors used for the creation of these device. The most common are amperometric, potentiometric, and voltametric sensor arrays [37]. E-tongues have seen lots of use in the evaluation of fish freshness, with different applications and results based on the characteristic of the sensors. Gil et al. [44] developed a device based on the use of a series of electrodes of different types (metal, metal oxide, and insoluble metal salt), for the evaluation of sea bream freshness, stored for a total of 14 days at 4 °C. An artificial neural network was used for the classification of samples into three temporal classes (first day, days 2–6, rest of days), obtaining a 90% correct classification. Barat et al. [45] published a paper regarding the same field of application, using an e-tongue composed of gold and silver wires. A high correlation was obtained between these measurements and ones made with reference methods, especially with K-value (R2 = 0.96), defined as the ratio between the sum of inosine and hypoxanthine to the sum of all other ATP breakdown products. On the other hand, Ruiz-Rico et al. [46] applied a voltametric e-tongue to cod samples, showing that the device could discriminate between fresh (days 0 and 1) and spoiled (from days 4 or more) fish samples, monitoring parameters like K-value and TVB-N (with an R2 value of 0.73 and 0.80, respectively). More recently, Miao et al. [47] applied a combination of the electronic nose and tongue for a post-cooking sensory evaluation of canned tuna. A PCA algorithm was used for the creation of a K-value calibration model, obtaining a satisfactory distribution of the samples by the first two principal components. Pattarapon et al. [48] used e-tongue, in conjunction with e-nose and several chemical parameters, to study the differences in grass carp quality between vacuum and non-vacuum storage. Results showed that with this device could differentiate between the three different storage conditions (vacuum 30 kPa, vacuum 50 kPa, and non-vacuum), supported by PCA and a linear discriminant analysis (LDA) analysis.

### 3.2. Colorimetric Sensor Array and Colorimetric Systems

The devices based on colorimetric sensor array technology monitor the process of chemical bonds creations (mainly metal and hydrogen bond, or π-π interaction) between different chemical agents [5]. The creation of these bonds affects the substances, shifting the absorption peak in the visible range, allowing an observation and quantification of the sample changes with the naked eye. An array of different dyes is commonly used to monitor several chemical substances at the same time. Image acquisition devices often help in quantifying the results, extracting the raw images, and processing them further [49]. Zaragoza et al. [50] created a chromogenic array for shelf-life assessment of fresh sea bream in cold storage, using a total of eight different sensitive materials. These data were then used as input for the creation of a PLSR model, with other data obtained from microbial and physico-chemical analyses. Recently, Morsy et al. [51] set up a device based on the responses of 16 dyes (like bromophenol blue and methyl red) to typical spoilage compounds like TVB-N, TMA, cadaverine, and putrescine, to monitor the spoilage process of Atlantic salmon. Dominguez-Aragon et al. [52] worked on a colorimetric method for the monitoring of tilapia fillets. This was accomplished thanks to the detection of color changes in copolymer substances, correlated with microbial growth and TVB-N. In the last couple of years, lots of papers regarding colorimetric sensing were published. Additionally, Zeng et al. [53] monitored TVB-N values, with a colorimetric indicator film based on mulberry anthocyanin extracts incorporated in a matrix. Tests on mud carp samples showed a correlation between the spoilage process and the color changes of the film, caused by the creation of volatile nitrogenous from the fish deterioration. Finally, Liu et al. [54] developed a colorimetric device based on filter paper, coated with a layer of sol-gel matrix entrapping a bromocresol green indicator, that monitors TVB-N levels. The matrix improved the moisture resistance of the system and allowed an accurate response to the fish freshness state. On the other hand, Valdez et al. [55] used an innovative technique, called “forcespinning”, to create nanofibers that have a strong electrostatic interaction with amine ions in the molecules of biogenic amines. The novel technique was tested with different types of meat (including fish), obtaining a detection limit of 7 ppm and a sensitivity of 40 counts/ppm for two biogenic ammines. Huang et al. [56] created a device for the monitoring of volatile compounds, based on agar incorporated with natural dye, testing it with Wuchang bream samples. The main results evidenced a great potential in terms of a “real-time” intelligent package system for convenient, non-destructive, and visual monitoring of fish spoilage.

Colorimetric systems differ from colorimetric sensor arrays in the subject of the investigation; instead of color changes strictly related to the creation of chemical bonds, they can analyze the samples without the need to induce chemical reactions. In fact, these devices are used to monitor changes in the whole sample, considering several visual and color parameters. For example, Quevedo et al. [57] set up a model based on lightness, redness, and yellowness values to assess the color of a salmon fillet. A high correlation (r = 0.95) with sensory panel methods was obtained. In another study, Dowlati et al. [58] monitored the ice storage of farmed and wild gilthead sea bream, investigating several color parameters, including lightness, redness, yellowness, total color change, and chroma. A high correlation (r > 0.95) was found between the parameters of the two fish species and storage days, thanks to the use of multiple regression models and ANN approaches. Balaban et al. [59] developed an image analysis method to follow the lightness value of snapper eyes stored on ice for seven days, focusing on the effects of polarized and non-polarized illumination, wet and dry surface, and contact or non-contact with ice. Similar studies were conducted on the gills of Indian rohu [60], pupil and gill of tilapia [61], and eye chromatic of European hake [62]. Finally, Taheri-Garavand et al. [63] proposed a system based on the use different statistical algorithms to analyze changes in the color parameters of common carp during storage in ice. Results showed that the ANN classifier had the best performance for fish freshness classification (93.01% accuracy). The presented electronic devices are shown in Table 2, which reports the type of application (measured parameters, fish species, place of application) and the statistical analysis algorithm and results.

**Table 2:** Electronic sensors.

System	Measured Parameters	Species	Place of Application	Statistical Analysis	R^2^/ Classification Rate (%)	Reference
Gas sensor array (FishNose)	TVC, Off Odor Rancid, LAB	Atlantic salmon	Both confined and open air	PLSR	94%	[38]
Integrated metal oxide micro-sensors	TVC	Sardine	Confined air (sampling vessel)	SVM/PLSR	0.91, 100%	[39]
Optical electronic nose	Bacterial growth	Tilapia	Confined air	-----	-----	[40]
MOS sensors (Mastersense)	TVC	Plaice, salmon	Confined air	KNN	92%	[41]
Metal oxide sensors	TVBN, bacterial counts	Hairtail fish	Confined air	PCA	0.91 0.97	[42]
Metal oxide sensors (UTS NOS.E)	Freshness state	Salmon	Confined air	Hidden Markov model	96%	[43]
Potentiometric electrodes	pH, TVBN, Microbial analysis	Sea bream	Contact with filet samples	MPL, PLSR	0.96	[44]
Au/Ag wires	K-value	Sea bream	Contact with sample solution	Least Square Method	0.96	[45]
Voltammetric sensors	K-value, TVB-N	Cod	Contact with filet samples	PLSR	0.79 0.73	[46]
Commercial e-tongue (ASTREE)	K-value	Canned tuna	Contact with sample filtrate	PCA	----	[47]
Commercial e-tongue (SA402B)	8 basic sense of taste	Grass carp	Contact with sample solution	----	----	[48]
Alluminium and silica-based materials	Microbial counts	Sea bream	Near to the sample (open air)	PLSR	0.92	[50]
Chemo-sensitive compounds	TVB-N, TBA, pH	Atlantic salmon	Near to the sample (confined air)	Linear fit	0.73 0.89 0.91	[51]
Copolymer substances	TVB-N, Microbial growth	Tilapia	Near to the sample (confined air)	----	----	[52]
Anthocyanin extracts	TVB-N	Mud Carp	Near to the sample (confined air)	----	----	[53]
Filter paper coated with a sol-gel matrix	TVB-N	Red drum	Fish package	Linear fit	0.97	[54]
Polydiacetylene nanofibers (ForceSpun)	Biogenic ammines	Not specified	Near to the sample	Linear fit	0.97	[55]
Agar with natural dyes	Volatile compounds	Wuchang sea bream	Near to the sample	PCA	87.5%	[56]
Computer vision system	Lightness, Yellowness, Redness	Salmon		----	----	[57]
Canon EOS digital camera	Gill and eye color changes	Gilthead sea bream		ANN	>96%	[58]
Nikon D300 digital camera	Eye lightness	Snapper		Excel correlation function	----	[59]
Nikon D90 digital camera	Gill color changes	Indian rohu		Statistical mean and STD calculation	----	[60]
Computer vision system	Gill and pupil color changes	Tilapia		Linear fit	>0.98	[61]
Nikon D7000 digital camera	Eye chromatic	European hake		PCA	r > 0.82	[62]
Computer vision system	Texture features	Common carp		SVM K-NN ANN	91.5% 90.5% 93.0%	[63]

## 4. Dielectric Techniques in the Radio Frequency Range

The study of changes in the dielectric properties can be considered a fast and non-invasive way to monitor different parameters of fishes. This method is based on the evaluation of the impedance Z from the Ohm’s law V = ZI, thanks to the use of two electrodes that apply a current flow I and a voltage V through the fish sample [64].

When an electric current is applied to the fish sample it can pass through two different types of pathways: one is a continuous extracellular fluid (ECF), considered to be purely resistive, whereas the second is a series of ECF and intracellular field (ICF) that includes the capacitive effect of the cell membrane, creating a complex impedance with a magnitude frequency dependence [64]. This impedance could be evaluated both as a mean value or as function of the frequency, as a spectrum.

Other than resistivity and conductivity, dielectric properties can be assessed by measuring the electrical permittivity, composed of two factors, the dielectric constant and the loss tangent. These two parameters are influenced by different food attributes, such as the moisture content [65], chemical composition [66], physical structure [67], water activity [68], and density [69]. Additionally, the frequency of the electromagnetic wave influences the results: if the temperature does not change, an increase in frequency causes a decrease in penetration depth [70].

The most investigated techniques are the open-ended coaxial probe, the transmission line, and the resonant cavity method. The first one consists in a coaxial line put in contact with the fish sample, measuring the reflection coefficient, related to the sample permittivity. The transmission line method is based on the use of an enclosed transmission line, filled with a solution of minced sample. Finally, for the resonant cavity method, a piece of sample, of a known geometry, is placed in a single-mode cavity, and the dielectric properties are computed from the changes in the reflected power and the frequency of resonance of the cavity [71].

An open-ended coaxial probe was used by Kent et al. [72] to assess several variables related to freshness on four different fish species: chilled and frozen Baltic cod (*Gadus morhua*), frozen Atlantic and Pacific hake (*Merluccius capensis* and *Merluccius australis*), and chilled farmed Atlantic salmon (*Salmo salar*). The measurement data were used as input for a PCA analysis, alongside other measured variables like the time of storage and temperature. Results showed that the age of whole gutted cod could be predicted with an error of 1.5 days, as well as the salmon one. The inclusion of measurements obtained with an electronic ‘nose’ sensor, as input for the PCA model, increased the precision up to ±1 day. Regarding hake, discrimination linear models were created for several variables, obtaining an R^2^ of 0.982 in validation, for the determination of an overall quality grade. Finally, discrimination models between different types and treatments of frozen fish (for hike and cod) were created, obtaining R^2^ values that were always higher than 0.83 in validation.

Wang et al. [73] focused on the effects of changes in temperature on the dielectric properties, monitoring five different frequencies (27, 40, 433, 915, 1800 MHz) in the temperature range of 20 °C–120 °C with an impedance analyzer based on a transmission line technique. The main results evidenced that, in the function of the temperature, the dielectric loss factor increased for each of the studied frequencies, whereas the dielectric constant had a dual behavior, increasing for 27 (from 97.8 at 20 °C to 149.6 at 120 °C) and 40 MHz but decreasing for 433 MHz (from 60.4 at 20 °C to 56.7 at 120 °C) and for higher frequencies. This reduction at microwave frequency was probably caused by the increase of the intermolecular vibrations that interrupted the water molecule arrangement. Additionally, the penetration depth was studied, observing a decrease of about five times between the HF-VHF (27 and 40 MHz) and the microwave range (915 and 1800 MHz). Finally, regression equations were calculated both for the dielectric constant and loss factor, for different temperatures and fillets parts, obtaining R^2^ values that were always higher than 0.977.

In 2008, Vaz-Pires et al. [74] characterized whole samples of cuttlefish (*Sepia officinalis*) and shortfin squid (*Illex coindetii*) stored in ice, with several analyses, including dielectric measurements carried out by two different fish freshness meters (Torrymeter type 295 by Distell Industries and RT-Freshmeter type RT-2E by Rafagnatækni Electronics). These devices are based on the presence of two pairs of concentrically arranged electrodes in a sensing head, applied directly onto the skin of the fishes. An alternating current is passed through the sample by the outer pairs of electrodes, whereas the inner pair senses the resulting voltage, acquiring in this manner measurements based on the skin impedance. Results showed that these instruments could be useful for the study of fish freshness, especially in the first days. Moreover, for both species, the less rigid areas were identified as the best sides for the acquisition: the ventral side for cuttlefish and the dorsal side for squid.

More recently, both studies conducted by Badiani et al. [75] and Rutkaiova et al. [76] using a fish freshness meter (Torrymeter type 295 by Distell Industries) studied changes in dielectric properties related to the icing of fishes. In particular, in the first article, these measurements were used for the prediction of the storage time and the setting up of discrimination tools between different icing treatments on cuttlefish. In contrast with the previous paper, dielectric properties did not show remarkable changes related to these two parameters, proving to be of little use for the detection of both fish freshness and the icing treatment used. In the second article, the fish freshness meter was used on samples of common carp (*Cyprinus carpio* L.) to develop a technique proving the falsification of thawed meat as fresh. The obtained results showed a correct classification of 100%, proving the utility of dielectric property measurement for this application. Table 3 shows a selection of the works dedicated to dielectric assessment, focusing on the type of application (studied parameters, fish species, and place of application).

## 5. Nuclear Magnetic Resonance Spectroscopy

Nuclear magnetic resonance (NMR) spectroscopy is based on the spin of atoms with an odd number of neutrons or proton, like hydrogen. Samples are placed in a magnetic field, where they are subjected to radio waves that excite the nuclei into resonance, detected with sensitive radio receivers.

This generated magnetic field changes the resonance frequency, thus giving access to details of the electronic structure of a molecule and its individual functional groups. As the fields are highly characteristic to individual compounds, NMR spectroscopy is particularly useful for the identification of monomolecular organic compounds. Usually, samples are dissolved in a solvent, because NMR analysis of solids requires dedicated components and may not give equally well-resolved spectra. Concerning fish applications, the most diffused NMR techniques are the low-field NMR (LF-NMR) and the high-resolution NMR (HR-NMR). The last technique appeared to be able in the discrimination between different species, geographical origin, production, and process history [37].

Ciampa et al. [77] studied the changes occurring on amino acids, main organic acids, and alcohols during Bogue fish storage for 15 days at 4 °C and on ice, focusing on the detection of hydrogen (^1^H-NMR). Results showed a slow increase of acidic amino acids and decrease of basic amino acids in the first days, due to protein autolysis, and a faster process afterward, due to microbial development. Moreover, it was observed that different storing temperatures cause differences in the amino acid trends starting at day 4. The storing process at different temperatures was also studied by Shulimina et al. [78,79] on salmon samples in two different studies, one focusing on temperatures of 0 and 4 °C and the other on 4 and 10 °C. In the first one, the HR-NMR technique allowed simultaneous measurement of the concentration of 31 different metabolites, including all the metabolites necessary for the evaluation of the K-index, obtaining results coherent with literature data. In the second one, NMR measurements were executed on an extract of salmon heads, backbones, and viscera, monitoring a total of 35 metabolites. A PCA analysis allowed the main chemical processes occurring during storage the maximum period of time for safe storage to be determined: seven days at 4 °C and three days at 10 °C. An evolution of the HR-NMR technique, called high-resolution magic angle spinning (HR-MAS), was tested by Heude et al. [80] to rapidly determine the K-value and TMA content from four different species (sea bream, sea bass, trout, and red mullet). There are three main advantages of this technique: it can be performed on unprocessed samples, it requires a quantity of fish (10–15 mg) compatible with industrial analysis, and it is characterized by a fast preparation of the samples, with the total analysis time in the order of 40 min. In the same year Jin et al. [81] assessed a different evolution of HR-NMR, the UltraFast intermolecular Single Quantum Coherence (UF iSQC), to analyze salmon muscle tissues, intact eggs from shishamo smelt, and a whole zebra fish. The main advantage of this technique is surely the speed of acquisition, allowing an analysis of the unprocessed sample in only 5 min. Additionally, Tan et al. [82] evaluated the freshness of intact zebra fish, using a high-resolution 2-D 1H J-resolved NMR technique that permitted the identification of 21 metabolites, and obtained their concentration variations during the spoilage process. With these data, pattern recognition models like PLS-DA and orthogonal partial least square discriminant analysis (OPLS-DA) were used to differentiate between fresh and decayed samples, for a total of five decay stages of spoilage (covering the range of 0–7 days). Regarding LF-NMR, Carneiro et al. [83] investigated the water mobility in the muscle of salted sardines on different days of storage. A significant correlation (*p* < 0.05) was found between the quality grade obtained by the chemical and physical parameters (cooking test, presence of ammonia and hydrogen sulfide) and the relaxation data obtained with NMR. Table 4 summarizes the recent works published on this technique.

## 6. Optical Spectroscopic Techniques

With the term “optical”, we refer to a family of techniques that use electromagnetic waves in the ranges of ultraviolet (UV), visible (VIS), and infrared (IR), to obtain information regarding the food freshness and quality. Over the years, it became an interesting and attractive research area thanks to the decrease of instrument prices, the improvement of equipment, and the combination with chemometric tools.

Regarding fish freshness, the main applications concern the detection of microbial growth and spoilage, the prediction of physicochemical and texture features, and the detection of issues due to different species and production methods. The main techniques taken into consideration are fluorescence spectroscopy, infrared spectroscopy, hyperspectral imaging, spectroscopy, and Raman spectroscopy. Table 5 summarizes a selection of the recent works published on this topic. 

### 6.1. Fluorescence Spectroscopy

The physical effect of fluorescence is related to the presence in the food samples of substances called “fluorophores”. They absorb energy from the external ambient, in the form of electromagnetic waves (usually from 180 to 800 nm); then, they emit this energy in the form of light, at a higher frequency [37]. Acquisitions are performed thanks to optical probes or dedicated spectrofluorometers. The fluorescence effect could be exploited with lots of different techniques that are mainly differentiated in the use of one single wavelength or a whole spectrum in the excitation of the fluorophores. Nowadays, one of the most interesting ones is synchronous fluorescence spectroscopy (SFS), where both exciting and emission wavelengths change at the same time, maintaining a positive difference. It allows information from several fluorophores to be obtained at the same time, considering the excitation emission matrix (EEM), which gives information on the whole three-dimensional fluorescence landscape [84]. Another well-known technique is front face fluorescence spectroscopy (FFFS); it solves problems due to a decrease in the fluorescence intensity and a distortion of the emission spectra, observed at a high absorbance rate [85].

The SFS technique was used by Liu et al. [86] to measure pyrene concentrations in the gills of carp fish. The technique was tested in the range of 1–1000 µgL^−1^, where it showed a linear behavior, and was compared to a more standard gas chromatography technique, obtaining a ratio of 1.003 between the measurements of the two techniques. In the same year, a classification of shrimp samples, according to their geographical origins and species, was performed by Eaton et al. [87] thanks to an EEM spectroscopy and SIMCA classification tool (3 incorrectly classified samples for both species and region, on a total of 22 and 24, respectively). In combination with PLSR regression models, EEM spectroscopy was explored by ElMasry et al. [88,89] in two different studies, both regarding the evaluation of horse mackerel samples. The freshness values were determined with a high-performance liquid chromatography (HPLC) and used for the creation of regression models, obtaining an R^2^ of 0.85 in the first work and up to 0.94 in the second one, where an optic fiber probe was used both for sample excitation and spectra capture, obtaining an EEM in the range of 250 to 800 nm. Fiber probes were also used by Shibata et al. [90] to develop a technique based on fluorescence fingerprint (FF) for the prediction of ATP content in frozen horse mackerel. The best PLSR model was found using only six excitation/emission wavelength pairs, with the major peak at 270/330 nm (R^2^ value = 0.88; RMSECV = 0.97 μmol/g).

Principal component analysis (PCA) was used in combination with the FFFS technique by Karoui and Hassoun [91] to study the differences between refrigerated, thawed then refrigerated, and refrigerated then thawed sea bass fillets. The best results were obtained with the excitation set at 380 nm, with an overall classification rate of 88.36%. Then, the whole data set was tested with a concatenation technique called common components and specific weights analysis (CCSWA), obtaining an overall classification rate of 94.9%.

More recently, Omwange et al. [92] studied the fluorescence emission of the fisheye of Japanese Dace at 365 nm, using a UV-LED. From these images, the color components were used to create a PLRS model for the prediction of K-value, obtaining an R^2^ value of 0.92 (RMSECV = 3.5%).

### 6.2. Infrared Spectroscopy

Spectroscopy in the near infrared and visible region (VIS/NIR) was historically explored in food analysis, due to the great presence of C–H, N–H, and O–H groups that vibrate in the VIS/NIR range of frequencies [93]. Measurements are usually performed thanks to spectrophotometers equipped with optical fibers or integrating spheres put in contact with the samples.

Tito et al. [94] researched FT-NIR spectroscopy in the range of 800–2500 nm to study the growth of microbial loads in Atlantic salmon over 9 days. The samples were investigated thanks to a fiber optic probe, and the mean spectra were used to create a PLSR model, obtaining an R^2^ = 0.95 (RMSEC = 0.12 log(cfu)/g). More recently, Reis et al. [95] explored the use of FT-NIR for the discrimination between fresh and frozen/thawed tuna samples. Spectra were obtained in the range of 300–2500 nm and used as input for a PLS-DA algorithm, obtaining a prediction ability of 92% for fresh samples and 82% for thawed ones.

In the last couple of years, deep learning techniques have been used more and more in this field: for example, Wu et al. [96] coupled FTIR spectroscopy (in the range of 400–1000 nm) with a stacked denoising autoencoder neural network (SDAE-NN) to predict the cold storage time of salmon, obtaining an R^2^ of 0.98 in prediction and a root mean square error in prediction (RMSEP) of 0.93 days whereas Agyekum et al. [97] studied the potentiality of a genetic algorithm to quantify volatile TMA concentrations in silver carps, using spectra acquired with an FT-NIR spectrometer and an optical fiber, resulting in an R^2^p of 0.980 (RMSEP = 5.1 mgN/100 g). A similar paper was published by the same research group in 2020, where FT-NIR was coupled with an ant colony PLS (ACO-PLS) algorithm to evaluate the K-value in silver carps, obtaining an R^2^p of 0.98 (RMSEP = 3.98). On the other hand, Alamprese et al. [98] explored the use of infrared spectroscopy (800–14,000 nm) in two discrimination problems: identification of different species (red mullet vs. Atlantic mullet, plaice vs. flounder) and discrimination between fresh and frozen thawed fillets of Atlantic mullet. Data were analyzed with two different algorithms, linear discriminant analysis (LDA) and SIMCA, obtaining sensitivity values of up to 83% and 97.2%, respectively. Another paper based on the application of chemometric algorithms coupled with IR (1000–1800 nm) was published by Zhou et al. [99]. Several parameters like pH and K-value were measured on bighead carp samples, and the predictive models were calculated with PLSR algorithms, coupled with a competitive adaptive reweighted sampling (CARS) for the selection of optimal variables. The best results were obtained for pH, with an R^2^ value of 0.95 and an RMSEP of 0.081, followed by thiobarbituric acid (TBA) (0.95/0.107), TVB-N (0.93/2.1), and K-value (0.81/6.5).

The mid-infrared (MIR) region of the electromagnetic spectrum (2500–25,000 nm) is one of the most informative sections of the whole electromagnetic spectrum. Like near-infrared (NIR), the use of Fourier transforms has significantly improved its capabilities and applications, replacing dispersive spectrometry. Hernandez-Martinez et al. [100] worked on FT-MIR in reflectance mode (2500–12,500 nm) to predict several physicochemical parameters in three different fish species (Atlantic bluefin tuna, crevalle jack, and Atlantic Spanish mackerel) (R^2^ between 0.92 and 0.98, PLSR models). More recently, Fengou et al. [101] combined FT-MIR (in the range of 2500–25,000 nm) with multispectral imaging (MSI) to monitor the microbial growth in sea bream. Results showed that FT-MIR gave better predictions for flesh samples (R^2^p = 0.73 and RMSEP = 0.72), highlighting that this technique could be more suitable for whole samples, with respect to fillets. Finally, Saraiva et al. [102] used a technique called attenuate total reflection (FTIR-ATR) to monitor, in the range of 5500–10,500 nm, the spoilage of salmon fillets at chilled temperatures in three different packaging conditions (R^2^ of 0.8 and 0.9, PLSR models).

### 6.3. Hyperspectral Imaging

The term hyperspectral imaging (HSI) refers to the blending of two different techniques, computer vision and VIS/NIR spectroscopy. With a hyperspectral camera it is possible to acquire a full spectrum for every pixel of an image, obtaining three-dimensional data called a “hyperspectral cube” [103]. After a pre-processing phase, a mean spectrum is calculated from every cube, and these are used as input for multivariate statistical analysis to resolve regression or classification problems. Finally, the calibration coefficients of the most informative wavelengths (often called “optimal” wavelengths) are used to calculate the parameters of interest for every pixel, creating a chemical map of the sample [104]. HSI techniques have been used for the development of fast and non-invasive analysis of fish freshness and overall quality, with three main application fields: chemical and physical analyses (moisture content, protein, or fat-related substances, textural features), monitoring of food processes (thermal processing, freezing and chilling, dehydration), and food safety evaluation (freshness evaluation, defect detection) [105].

Cheng et al. [106] studied the use of HSI in the range of 400–1000 nm to evaluate the K-value in grass carp and silver carp. PLSR and least square support vector machine (LS-SVM) were used to create regression models, both with full spectra and with only seven optimal wavelengths selected by a successive projection algorithm (SPA). The best results were obtained with the full-wavelength PLSR, with an R^2^p (in prediction) of 0.94 (RMSEP = 5.21%). In 2016, the same research group expanded the previous work, using LS-SVM and multiple linear regression (MLR) to simultaneously determine the K-value, TVB-N, and thiobarbituric acid reactive substances (TBARSs), with the support of SPA and genetic algorithm (GA) for the determination of the optimal wavelengths [107]. Both algorithms gave excellent results in the determination of K-value and TVB-N, with an R^2^p > 0.90, with five wavelengths found by SPA and six wavelengths found by GA. In 2017, they combined the GA with an innovative algorithm called physarum network (PN), focusing on the determination of the TVB-N [108]. PLSR and LS-SVM models were created with the six optimal wavelengths found by the PN-GA algorithm. The use of image texture variables, extracted by gray-level gradient co-occurrence matrix (GLGCM), enhanced the model, obtaining an R^2^p of 0.98 (RMSEP = 1.44 mgN/100 g). In 2016, Ivorra et al. [109] developed a model for shelf-life prediction of salmon in two different steps: first, they used a K-nearest-neighbor model to classify the fish tissue into fat and muscles, and then they created a PLSR model for each group (400–1000 nm). The model created with only fat tissue spectra obtained the best results, with an R^2^_CV_ (in cross-validation) of 0.89 (RMSECV = 2.7 days). More recently, Khoshnoudi-Nia et al. [110] studied the assessment of TVB-N, psychotropic plate count (PPC), and sensory score in rainbow trout fillets. Four different multivariate analyses were developed: PLSR, MLR, LS-SVM, and back propagation neural network (BP-NN), each of which used six optimal wavelengths determined by GA, coming from the original range of 430–1010 nm. All models showed good performance in the prediction of all three parameters (R^2^p ≥ 0.856), with the best results coming from LS-SVM (R^2^p = 0.91). 

The HSI technique could be used to measure parameters not related with freshness. For example, Xu et al. [111] worked on the assessment of gross energy density values in salmon fillets. In this work, images were acquired in the range of 900–1700 nm and used for the creation of PLSR and epsilon-SVR models. Altogether, the best results were obtained with a stepwise-PLSR model (R^2^p = 0.908 and RMSEP = 6.87%), where only four wavelengths (931, 1001, 1135, and 1168 nm) were selected. Another example is provided by the work conducted by Qu et al. [112] to map the moisture content in grass carp fillets subjected to different freeze-drying conditions. The spectra (400–1000 nm) were used as input for a PLSR model, and the application of regression coefficients and weighted values resulted in the selection of nine optimal wavelengths, with a final R^2^p of 0.91 (RMSEP = 56.3 g/kg). Finally, Qin et al. [113] studied the detection of fillet substitution and mislabeling (fresh/thawed), using reflectance in the VIS/NIR region (419–1007 nm), fluorescence by 365 nm UV excitation (438–718 nm), reflectance in the SWIR region (842–2532 nm), and Raman by 785 nm laser excitation (103–2831 cm^−1^). Images were acquired on six different fish species (red snapper, vermilion snapper, Malabar snapper, summer flounder, white bass, and tilapia), and used as input for 24 different machine learning techniques. In general, the VIS/NIR reflectance mode gave the best performance for both species and freshness inspection, proving to be the best wavelength range for the detection of fish fillet substitution and mislabeling.

### 6.4. Raman Spectroscopy

Raman spectroscopy is based on the measurement of the vibrational energy modes thanks to the use of scattered light: A monochromatic laser beam irradiates the sample (UV, VIS or NIR), causing a shift in the vibrational energy level of the molecules from the ground state to a state of high-energy collision. When these molecules quickly return to the ground state, the physical process of Raman scattering occurs, allowing structural and chemical information of the sample to be obtained at the same time [114]. Measurements are performed with dedicated Raman spectroscopy, starting from minced samples put into solution. Sanchez-Alonso et al. [115] showed that the alteration in the spectra related to lipid oxidation was in the Raman stretching wavenumber regions of 1658 cm^−1^ in frozen hake fillets. Janci et al. [116] evaluated a technique called surface enhanced Raman spectroscopy (SERS) to determine the histamine content in Atlantic mackerel, in the range of 1050–1650 cm^−1^. The resulting spectra were used as input for a PLSR analysis (R^2^p of 0.97 and RMSEP of 14.9 mg/kg). Histamine content was also the parameter analyzed by Xie et al. [117] that used SERS on thin-layer chromatography plates containing fish samples, with an innovative use of silver nanoparticles in conjunction with an NaCl aqueous solution, suppressing the masking effect caused by excessive fluorescamine (a reagent used for the detection of amines and peptides). The spectra were acquired in the range of 600–1800 cm^−1^ and compared with the results of a more traditional, but less cost and user-friendly, HPLC technique, obtaining good agreement and reproducibility. Another application of Raman spectroscopy is represented by the detection of unwanted chemical products in fishes. Deng et al. [118] investigated the use of SERS for the detection of Green Malachite (GM) in fish samples, by means of a self-developed glass fiber paper as substrate for the samples. Spectra were acquired in the range of 300–1700 cm^−1^, and an R^2^ of 0.99 calculated in the range of 10^−7^–10^−5^ mol/L, with an LOD of 20 ng/mL, was observed.

Regarding classification problems, Velioglu et al. [119] investigated the effects of freezing on fishes with Raman spectroscopy, to discriminate between fresh, once frozen-thawed, and twice frozen-thawed. The analysis was carried out on six different species (horse mackerel, European anchovy, red mullet, bluefish, Atlantic salmon, and flying gurnard), in the range of 200–2000 cm^−1^. The spectra were analyzed with a PCA that allowed successful discrimination between the different classes. More recently, Song et al. [120] developed a classification model for the detection of fish bones in grass carp fillets, analyzing them with a Raman hyperspectral imaging system in the range of 820–2847 cm^−1^. An algorithm called the fuzzy-rough set model based on the thermal-charge algorithm (FRSTCA) was applied to the resulting spectra to select the spectral band containing the major amount of information, which was 961–965 cm^−1^.

From these data, a support vector data description (SVDD) classification model was established, obtaining a detection performance of 90.5%. Table 5 summarizes the presented spectroscopic techniques, focusing on the type of application (measured parameters, fish species) and on the statistical analysis (algorithm, statistical results).

**Table 5:** Spectroscopic techniques.

Spectrocopic Technique	Measured Parameters	Species	Statistical Analysis	Best R^2^/ Classification Rate (%)	Reference
SFS	Pyrene	Carp	-----	-----	[86]
EEM	Geographical origins Species	Shrimp	SIMCA	91.7%	[87]
EEM	K-value	Horse mackerel	PLSR	0.89/87.5%	[88]
EEM	Freshness value	Horse mackerel	PLSR	0.94	[89]
Fluorescence Fingerprint	ATP content	Horse mackerel	PLSR	0.88	[90]
FFFS	Thawing/Refrigerating process	Sea bass	CCSWA	94.9%	[91]
UV-LED system	K-value	Japanese Dace	PLSR	0.92	[92]
NIR (800–2500 nm)	Growth of microbial load	Atlantic salmon	PLSR	0.95	[94]
NIR (300–2500 nm)	Fresh/thawed	Tuna	PLS-DA	92%	[95]
NIR (400–1000 nm)	Cold-storage time	Salmon	PLSR, BP-NN, SDAE-NN	0.98 (SDAE-NN)	[96]
NIR/MIR (800–14,000 nm)	Species Fresh/thawed	Red mullet, Plaice Atlantic mullet, Flounder	LDA, SIMCA	97.5%	[98]
NIR (1000–2500 nm)	TMA concentration	Silver carp	PLSR, GA-PLS	0.98 (GA-PLS)	[97]
NIR (1000–1800 nm)	K-value TVB-N TBARS pH	Bighead carp	PLSR	0.81 0.93 0.95 0.95	[99]
FT-MIR (2500–25,000 nm)	Chemicophysical parameters	Atlantic bluefin tuna, Crevalle jack, Atlantic Spanish mackerel	PLSR	>0.92	[100]
FT-MIR (2500–25,000 nm)	Microbial growth	Sea bream	PLSR	0.73	[101]
FTIR-ATR (5500–10,500 nm)	TVC	Salmon	PLSR	0.8	[102]
HSI (400–1000 nm)	K-value	Grass and silver carp	PLSR, LS-SVM	0.94 (PLSR)	[106]
HSI (400–1000 nm)	K-value TVB-N TBARS	Grass carp	LS-SVM MLR	0.93 (MLR) 0.87 (LS-SVM) 0.94 (MLR)	[107]
HSI (400–1000 nm)	TVB-N	Grass carp	PLSR LS-SVM	0.98 (PLSR)	[108]
HSI (400–1000 nm)	Shelf-life prediction	Salmon	PLSR	0.89	[109]
HSI (430–1010 nm)	TVB-N PPC Sensory score	Rainbow trout	PLSR, BP-NN, LS-SVM, MLR	0.91 (LS-SVM)	[110]
HIS (900–1700 nm)	Gross energy density	Salmon	PLSR, epsilon-PLSR	0.91 (both)	[111]
HSI (400–1000 nm)	Moisture content	Grass carp	PLSR	0.91	[112]
HIS (400–2500 nm)	Species Fresh/thawed	Red snapper, Vermilion snapper, Malabar snapper, Tilapia white bass, Summer flounder	24 different ML algorithms	100% (VNIR) 99% (SWIR)	[113]
FT–Raman (900–4000 cm^−1^)	Lipid content	Hake	---	----	[115]
SERS (1050–1650 cm^−1^)	Histamine content	Atlantic mackerel	PLSR	0.96	[116]
SERS (600–1800 cm^−1^)	Histamine content	Ribbonfish, Tuna	----	----	[117]
SERS (300–1700 cm^−1^)	Green malachite	Not specified	----	----	[118]
Standard Raman (200–2000 cm^−1^)	Fresh/thawed	Horse mackerel, Flying gurnard red mullet, European anchovy, Atlantic salmon, Bluefish	PCA	----	[119]
Raman HIS (820–2847 cm^−1^)	Detection of fish spines	Grass carp	SVDD	90.5%	[120]

## 7. Conclusions and Future Trends

The main families of innovative systems for fish freshness assessment and spoilage monitoring were reviewed. Biosensors, electronic sensors, dielectric devices, and spectroscopy techniques were covered, with a secondary focus on the several algorithms (multivariate statistical analysis, or more complex machine learning and deep learning techniques) used for the set up of classification/regression models and chemical maps. Some of these devices, thanks to a fast and less invasive acquisition process, are much more suitable for in-line or on-line industrial monitoring with respect to traditional methods (sensory, physical, and chemical parameters), which are costly, time consuming, and require high-skill operators. Spectroscopic devices could represent the best method for industrial application, being compact and not requiring contact with the fish. Between these techniques, hyperspectral imaging could be the most interesting for the huge amount of gathered information, although the acquisition speed of hyperspectral cameras must be increased before it can be used in practice. To obtain this important improvement, algorithms for the selection of few optimal wavelengths should be applied, optimizing and speeding up the measurement phase. Finally, biosensor applications in the food package could represent a good freshness monitoring method for products on the market.

However, the main conclusion that can be reached by the review of the literature is that, nowadays, all the described techniques have specific advantages and disadvantages: none of them can perform a satisfactory measurement of the overall fish quality by themselves. For this reason, the mentioned devices have not yet had extensive and widespread use in practical applications. In addition, several of the examined solutions are destructive. So, in the near future, research could be conducted on the combination of different techniques, allowing an increase of the volume of information extracted from the sample and the limitations of each individual method to be overcome. The continuous innovations in computer science and in complex statistical analysis could allow the use of these techniques, especially the spectroscopic ones, in an increasing number of different applications.

## Figures and Tables

**Table 1:** Analytical and chemical biosensors.

System	Measured Substance	Species	Application	LOD	Detection Range	Reference
Amperometric enzymatic biosensor	Xa	Crucian carp	Contact with sample solution	20 nm	0.03–21.19 µM	[21]
Ammonium ion-selective electrode	Volatile ammines	Cod	Package atmosphere	0.01 ppm	1–250 ppm	[24]
Amperometric enzymatic biosensor	Xa	Channa striatus	Contact with sample solution	2.5 pM	0.4–2.4 nM	[30]
Acqueous phase electrode	Conductivity/pH	Cod	Package atmosphere	-----	-----	[25]
Amperometric enzymatic biosensor	Xa	Labeo fish	Contact with sample solution	0.5 µM	0.5–500 µM	[31]
Amperometric enzymatic biosensor	Xa	Hake	Contact with sample solution	13 nM	0.05–12 µM	[32]
Amperometric enzymatic biosensor	Hystamine	Carp, Prussian carp, Tench, Wels catfish, European perch	Contact with sample solution	25.4 nM	0.1–300 µM	[22]
Organic gas sensor	Volatile ammines	Tilapia, Beltfish, Mackerel	Air pushed in the sensor chamber	0.1 ppm	0.1–1 ppm	[26]
Graphite electrode	HxA Xa UA	Barracuda, Lady fish, Mackerel, Blue cat fish, Channel cat fish	Contact with sample solution	----	6–30 µM 8–36 µM 3–21 µM	[27]
Pyrolitic graphite electrode	HxA Xa UA	Tuna, Hake, Myleus paku, Silverside	Contact with sample solution	0.08 µM 0.06 µM 0.03 µM	0.1–50 µM 0.1–50 µM 0.1–25 µM	[33]
Carbon electrode	Histamine	Spiked tuna, Mackerel	Contact with sample solution	0.97 mgL^−1^	----	[34]
Copper phosphate electrode	Histamine	----	Contact with sample solution	3.0 ppm	5–500 ppm	[28]
Cu-BTC framework	HxA Xa	----	Contact with sample solution	----	0–10 µM 0–8 µM	[29]
Glassy carbon electrode	Xa	Salmon	Contact with sample solution	0.35 nM	0.001–50 µM	[35]
Fluorescent biosensor	HxA	Fish, Shrimp, Squid	Contact with sample solution	2.88 µM	8–2500 µM	[36]

**Table 3:** Dielectric techniques.

System	Studied Parameter	Species	Place of Application	Reference
Open-ended coaxial probe	Storage time	Baltic cod, Atlantic hake, Pacific hake, Atlantic salmon	Contact with sample	[72]
Impedance analyzer	Dielectric properties and penetration depth	Salmon	Cylindrical samples placed in a test cell	[73]
Fish freshness meter	Fish freshness	Cuttlefish, Shortfin squid	Contact with sample	[74]
Fish freshness meter	Storage time, icing treatments	Cuttlefish	Contact with sample	[75]
Fish freshness meter	Dielectric property changes for different icing treatments	Common carp	Contact with sample	[76]

**Table 4:** Nuclear magnetic resonance techniques.

Spectroscopic Technique	Measured Parameters	Species	Reference
^1^H-NMR	Amino acid, organic acid, alcohols	Bogue fish	[77]
^1^H-NMR	K-value	Salmon	[78]
^1^H-NMR	Maximum storage time	Salmon	[79]
HR-MAS	K-value TMA-N	Sea bream, Sea bass trout, Red mullet	[80]
UF–iSQC	Amino acids Fatty acids	Salmon, Shishamo zebra fish	[81]
^1^H 2D J-resolved NMR	Metabolites content	Zebra fish	[82]
LF-NMR	Water mobility	Salted sardines	[83]

## Data Availability

Not applicable.
